# Supplementary material for: Variance in odds ratios for estimating the deterrent effect of darkness on cycling: Variation due to the choice of case and control hours
Source: PLoS One. 2024 Oct 11;19(10):e0311964. doi: 10.1371/journal.pone.0311964 (PMC11469482; doi:10.1371/journal.pone.0311964)
Supplement: Attachment — Submitted filename: Response to Reviewers.docx [file pone.0311964.s004.docx]

**PONE-D-24-14676**

Variance in odds ratios for estimating the deterrent effect of darkness on cycling: Variation due to the choice of case and control hours

Response to review comments

| # | Comment | Authors’ response |
| --- | --- | --- |
| 1 | Reviewer #1: Thank you to the editor and the authors for the opportunity to review this paper entitled “Variance in odds ratios for estimating the deterrent effect of darkness on cycling: Variation due to the choice of case and control hours. This study investigated the impact of the choice of case and control hours on ORs obtained for cyclist flows in different countries.  The idea of this paper is interesting, and it seems to be important to help the knowledge of the area. Thus, below are some points that, in my opinion, would help the authors increase the quality of the study. | n/a |
| 2 | ABSTRACT In line 17 is the first moment the word “odds ratios” appears, but there is no acronym description, OR, however, in line 20, the acronym ORs appears. So, please, insert the acronym in the first description of the odds ratios. | Agree: Abbreviation added. |
| 3 | Additionally, it would be important, that the authors insert the “p values” in the abstract, rather than only describe “…the variance was larger… (line 21)”, or “…this suggested a statistically significant deterrent… (line 25)”. | Agree: P-value added for the final conclusion. |
| 4 | INTRODUCTION. I suggest the authors remove or reorder (ex. supplementary document or only citing the study) both tables 1 and 2 from the introduction. The way they were inserted affects the fluidity of reading. | We would prefer to retain these Tables in the main body of the manuscript. The disruption to reading flow suggested by the reviewer is not likely to be present in the formatted article. |
| 5 | METHODS  Line 175. There is a double “that” written. | Agree: The second instance is changed to ‘the’. |
| 6 | Why did the authors use eight years in the new locations rather than four years? And, why choose different years? Could these differences in the years impair the analysis? | For those cities used in previous studies we used for consistency the same 4-year periods as were used in the original studies. For the two new cities we planned to use larger data ranges, with the caveat of stopping at the end of 2019 to avoid any confound following travel changes due to Covid. For Leeds there are data available from 2012. For Berlin, data were not available until 2016.  For Leeds we used the 8-year period 2012 to 2019. Analysing each of the successive 4-year periods in that range produced ORs ranging from 1.15 to 1.33. Using any of these would not have changed our conclusion about significance of the deterrent effect of darkness. |
| 7 | DISCUSSION Why were the formulas inserted in the discussion? The discussion highlights the study's important findings, pertinent conclusions, and their implications or applications. So, it would be better to transfer the formulas for the Method session. | We disagree with this suggestion to move equations 3-5. The focus of this work is whether different combinations of case and control hour lead to different ORs. The results therefore show the variance in ORs, and it is that variance which led to the need for establishing a weighted mean. We would prefer to introduce the weighted OR equations (3, 4 and 5) in the discussion, i.e. introduced after recognition of the variance in ORs for discrete case and control hours. However, we will move these if requested by the editor. |
| 8 | While in the introduction, the authors talk about the influence of the weather on the flow of cyclists, in the discussion, the topic is not presented. I think that the authors would invest in this topic. | We raise weather in the introduction to demonstrate awareness of this factor, but weather is not part of the scope of this article (which is the choice of case and control hours) and thus it is not included in the discussion. |
|  | **Comments from the editor** |  |
| 9 | Please make sure that the structure for citing published literature in the text, as well as the style of references in the References section, are consistent with the journal's style (see Instructions to Authors). | The reference list has already been organised according to the journal's style (Vancouver). |
| 10 | English language needs revision for style and syntax. | The authors include two native English speakers who are also journal editors and consider the language to be correct. We would be pleased to give attention to specific issues the editor points out. |
| 11 | Abstract must be rewritten. I suggest focusing the abstract on your study and your results. | The opening sentences have been rephrased to focus only on cyclist flows |
| 12 | Please add the originality of the study and add hypothesis at the end of the introduction section. Be please be more specific. | We added the following text within section 1: “*This study is the first to examine whether the researchers’ choice of case and control hours affects the outcome of analyses investigating the impact of ambient light levels on traffic flow. We test two hypotheses: that darkness deters cycling, as indicated by ORs >1.0, and that varying the choice of case and control hours will lead to different ORs*.” |
| 13 | Include more characteristics of participants. More information about the participant’s selection needed. | There were no direct participants. The data used in this analysis were drawn from automated traffic counters. |
| 14 | Results are not clear. I would only appreciate to read a detailed statistical approach | As with comment 13 we are not sure this is really specific to our study. We state the approach to testing significance in section 2.2. It is not usual to describe in detail the nature of statistical tests and included a reference where such detail can be found. |
| 15 | Please discuss the results of the study in relation to the previous studies. | This is done in section 4.3, titled “Comparison with previous studies” |
| 16 | Add the public implications of this study. | Text added to the end of the conclusion:  “*Evidence that darkness has a deterrent effect on cycling provides support for the use of road lighting after dark to encourage people to cycle rather than to use motorised transport or to avoid travelling. This in turn supports global initiatives to promote active travel and this improve the sustainability of travel.*” |
