# Supplementary material for: Variance in odds ratios for estimating the deterrent effect of darkness on cycling: Variation due to the choice of case and control hours
Source: PLoS One. 2024 Oct 11;19(10):e0311964. doi: 10.1371/journal.pone.0311964 (PMC11469482; doi:10.1371/journal.pone.0311964)
Supplement: Attachment — Submitted filename: Response to Reviewers.docx [file pone.0311964.s005.docx]

**PONE-D-24-14676**

Variance in odds ratios for estimating the deterrent effect of darkness on cycling: Variation due to the choice of case and control hours

Response to review comments

| # | Comment | Authors’ response |
| --- | --- | --- |
| 7 | Please, addresses the recommendation raised by the reviewer on item 7 (regarding the equations cited in the discussion). Please, remove the equations to "methods section" if they were used in the study's procedures or to a more suitable place (tables or supplementary material if applicable). | In the methods section, we present two equations (1 and 2) used to determine odds ratios (OR) and associated confidence intervals. This is done for each combination of case and control hours, leading to five matrices of ORs (Tables 4 to 8). It is only after detecting the variance in each matrix that the need for a weighted mean becomes apparent, and the equations (3, 4, and 5) necessary for that are thus presented after the results, in the discussion section.  We would prefer to keep equations 3 to 5 in the discussion section, as this better maintains the flow of information.  Therefore, no changes have been made to the document. |
